# Notes on a Case of Spina Bifida

**Published:** 1829

**Authors:** 


ART. V.—*Notes on a case of Spina-Bifida.*—By THE EDITOR.

I am indebted to Dr. Bradbury, of this city, for the opportunity of becoming acquainted with the history and pathological anatomy of a recent case of Spina Bifida, some account of which is, perhaps, worthy of publication.

The patient, an infant daughter of G. G. a respectable mechanic, was born with a tumour near the junction of the lumbar vertebræ with the sacrum. It was nearly of the shape and size of half a hen's egg, when divided longitudinally. Regarding it as Spina Bifida, Dr. Bradbury directed a bandage round the body of the child, which was employed, as I understood, without a compress or pad to cause pressure, especially over the tumour; this continuing to grow, the bandage was at length laid aside. When I saw it, about the middle of August, four months after birth and three days before its death, the swelling was near three inches and a half high and two and a half in diameter; and resembled, in figure, a tin cupping vessel. Its parieties were vascular, of a rose color, semi-transparent, and in places extremely attenuated. The fluid which it contained would not recede on pressure.

Over the anterior fontanelle, and thence to the centre of the lower part of the os frontis, there was another congenital projection, which consisted of a fluid confined beneath the scalp. It was much less prominent than the spinal tumour, having grown at a different ratio.

The general health of the little patient had not been bad, though I found it considerably emaciated. It had suffered a great deal of pain, particularly on being moved; and its mother had given it laudanum, daily, as a palliative. At first this medicine rendered it costive; but subsequently its bowels became regular, and its digestion was good. Its appetite was natural and it seldom vomited. When, not in pain, nor stupified by laudanum, it was cheerful and exhibited all the intelligence usual at its age. The sensibility of its lower extremities was unimpaired; and their motion rather greater than common. As to strength, it had sufficient to bear its own weight. It never had convulsions, nor any degree of paralysis in any part.

The secretion of urine was habitually deficient. When the tumour was compressed, after it had attained to a considerable size, an alvine dejection would immediately follow.

Two days before its death a spot of the cyst was so far absorbed, that the contents began to weep away, and the integuments to shrink up. This discharge was attended with an abatement of the swelling on the head.

The phenomena which preceded its dissolution, were described as being those of debility, without spasmodic action of any kind.

*Post mortem examination.*—The cranial tumour subsided when the spinal was punctured, and its contents suffered to escape. The scalp was not laid open, but it was easy to perceive, that great deficiencies existed in the anterior part of each half of the os frontis, constituting a wide and irregular chasm, from the front end of the sagittal suture to the ossa nasi. Through this elongated opening, either the brain must have protruded from the pressure of serum in the ventricles; or the dura mater only, from an accumulation, beneath it, of the cephalo-spinal fluid of Majendie.

On examining the sacro-lumbar cyst, it was found considerably shrunken and its parieties corrugated. The discharge from it was somewhat turbid, and a little offensive. The mother stated, that the first which issued on the bursting of the cyst, was of a green color. We removed the entire sack with the implicated portion of the spine. The aperture was the consequence of a deficient ossific action in the posterior part of the two lowest lumbar vertebræ, the spinous processes of which were wanting. The opening was large enough to admit the thumb. The effusion was on both sides of the arachnoid. The spinal marrow had been drawn, in duplicature, through the bony aperture, so that when the sack was distended, it must have projected through that cavity quite to its outer extremity. Thus the origins of the cauda equina had been dislocated—they had, in fact, been dragged out of vertebral canal. This singular displacement arose from a morbid adhesion of the membranes of the spinal marrow to the integuments at the apex of the cyst, a union, occasioned no doubt, by the inflammation which the pressure applied soon after birth had excited; and which, when subsequent effusion took place, with a consequent elevation of the integuments, enabled them to carry with them a duplicature of the medulla spinalis, nearly in the manner that it might have been drawn out, by passing a ligature around it, and pulling towards the apex of the cyst. A portion of nervous matter could be traced quite to the integuments, but it was greatest near the aperture.

The membranes throughout the whole extent of this conical projection, but especially towards its apex, were in a state of inflammation—exhibiting a florid hue and masses of coagulable lymph.

To this inflammation, we should doubtless ascribe the sufferings of the child, and the locomotive propensity of the muscles of its lower extremities. That they should, under such a condition of the origins of their nerves, have retained their sensibility, and remained free from paralysis or spasms, are facts that deserve at least to be noted. The constant effect of pressure on the cyst in occasioning immediate alvine evacuation, is likewise worthy of preservation.

*September*, 1829.

